# Social determinants of health in medical education: insights from final-year students in a multicentre study in Italy

**DOI:** 10.1007/s13304-026-02619-5

**Published:** 2026-04-07

**Authors:** Giulia Milanesi, Basem A. Khalil, Antonino Morabito

**Affiliations:** 1 University of Florence, Florence, Italy; 2 King’s College Hospital – London, Dubai, UAE; 3 University of Florence, Florence, Italy; 4 Department of Paediatric Surgery, Center for Intestinal Reconstruction and Rehabilitation, Meyer Children’s Hospital IRCCS, Florence, Italy; 5 Department of NeuroFarBa, University of Florence, Florence, Italy

**Keywords:** Health equity, Medical education, Public health, Social determinants of health, Italy

## Abstract

Social determinants of health (SDH) are fundamental to understanding health inequalities. However, the extent of their integration into Italian medical education remains underexplored. A questionnaire based multicenter study was conducted among 30 final-year medical students chosen through purposive sampling from three Italian universities: University of Florence, Humanitas University Milan, and University of Naples. Data were analyzed using both quantitative and qualitative approaches. Students displayed partial theoretical awareness of SDH but limited applied understanding. Differences across regions reflected institutional approaches. Education, socioeconomic status, and diet were perceived as the most relevant determinants. Findings highlight the need to strengthen SDH teaching in medical curricula to improve future physicians’ capacity to address health inequities.

## Introduction

Social determinants of health (SDH) have been defined by the World Health Organization (WHO) as the non-medical factors that influence health outcomes, encompassing the conditions in which people are born, grow, work, live, and age, as well as the broader forces and systems shaping daily life. These conditions are driven by political, social, and economic factors [1, 2].The SDH framework therefore addresses both the determinants of health and the origins of health inequities [3]. It has been estimated that SDH exert a more significant influence on population health than medical care, accounting for approximately 30–55% of overall health outcomes [4].

The understanding of SDH has evolved. In the nineteenth century, Rudolf Virchow emphasized the role of poverty and living conditions in disease spread, initiating the concept that health is shaped by social context [5]. In Italy, scholars such as Bizzozero contributed to the establishment of early public hygiene systems and advanced the notion of collective responsibility for public health [6]. During the 1970s, public health perspectives expanded with the Lalonde Report, which recognized biological, environmental, lifestyle, and healthcare organization factors as key components of health [7]. Subsequent milestones included the 1978 Alma-Ata Declaration, advocating primary health care and health equity as fundamental human rights [8], and the 1986 Ottawa Charter, which consolidated health promotion principles and highlighted the role of peace, education, income, social justice, and equity as prerequisites for health [9–13]. Later contributions, including the work of Michael Marmot and the WHO Commission on Social Determinants of Health, have refined the modern SDH framework into nine core domains [14–17].

Among these, education, economic stability, social support, and environmental context have been consistently recognized as central to health and well-being [18–24]. Education, in particular, profoundly shapes employment opportunities, income, health literacy, and behaviors, and inequities in access to education have long-term effects on morbidity and life expectancy [18, 19]. Likewise, social and community networks, employment conditions, and access to healthcare all contribute to disparities in population health [20–23].

Despite the global emphasis on SDH, its integration into undergraduate medical education remains limited. Medicine has advanced substantially in understanding biological mechanisms of disease, yet social and contextual factors continue to be insufficiently addressed in medical curricula [38–41]^.^ In Italy, the extent to which SDH are taught during medical training has not been systematically assessed. Therefore, this multicenter study involving medical schools from three different Italian regions (North, Center, and South) aimed to evaluate the level of understanding of SDH among final-year medical students and to explore how these topics are incorporated into university curricula.

## Materials and methods

A total of thirty final-year medical students were enrolled using a purposive sampling approach. Participants were recruited from three Italian universities representing distinct geographical areas: ten from the University of Florence (Central Italy), ten from Humanitas University in Milan (Northern Italy), and ten from the University of Naples Federico II (Southern Italy). At the University of Florence and the University of Naples Federico II, the medical degree program is delivered in Italian, whereas Humanitas University offers an international medical curriculum taught entirely in English.

All participants were enrolled in the standard six-year medical degree program of their respective institutions.

All students included in the study were of Italian nationality.

The sample was evenly balanced by gender across institutions, with an equal distribution of female and male students within each university cohort (five women and five men per institution).This multicenter design was intended as an initial exploration of potential regional variations in students’ understanding of social determinants of health (SDH).

The complete questionnaire is presented in table form to maintain clarity and consistency (see Table 1).

**Table 1:** The questionnaire

1.	What is your understanding of social determinants of health?	
	a.	What social determinants of health do you remember?
	b.	How do you think these influence, or affect the health of individuals and can you give examples?
2.	Which of the social determinants of health you mentioned do you think is the most important?	
	a.	Why do you think this is the most important?
	b.	How do you think this particular determinant could be used to improve population health?
3.	Do you think there are enough investments in social determinants of health at the national government level?	
	a.	Do you know of any national government campaigns targeting any of the social determinants of health?
	b.	Do you think hospitals are given preferentially more funding than the social determinants of health?
4.	Do you think there is enough education in the medical curriculum on the importance of social determinants of health?	
	a.	Did you receive any lectures specifically looking at the social determinants of health? If not, how did you know about them?
	b.	Do you think medical doctors should be at the forefront of advocating for the social determinants of health?
5.	Have you heard of the WHO Ottawa Charter 1986?	
	a.	If yes, where did you hear about it?
	b.	Are you aware of any international goals, agreements, or treaties regarding any of the social determinants of health?

The interviews were conducted from June 2023 to January 2024, either face-to-face or remotely, depending on participants’ availability. The majority took place via video or phone calls to facilitate participation across distances. All students provided informed consent before taking part in the study. Each interview was audio-recorded and subsequently transcribed verbatim for analysis.

Data analysis adopted a mixed qualitative–quantitative approach. Thematic content analysis was first conducted separately for each university to identify local patterns and contextual differences. Subsequently, findings were synthesized across institutions to highlight common themes and interregional variations. Quantitative elements, such as frequency of recurring topics, were integrated to complement qualitative insights and ensure analytical rigor.

This dual-level analysis allowed the researchers to capture both the specific experiences and perspectives unique to each institution and the broader trends emerging across the collective dataset.

## Result

The present study investigated the understanding of social determinants of health (SDH) among final-year medical students from three Italian universities (Florence, Milan, and Naples). Data were derived from interviews, integrating quantitative findings with qualitative thematic insights.

### General understanding of SDH

Most students demonstrated a basic awareness of SDH, although definitions often required prompting. A small minority articulated a comprehensive understanding, while the majority recognized the existence of both proximal and distal determinants influencing health outcomes.

Across universities, comprehension levels were similar. In Florence and Naples, students were equally divided between those needing prompting and those showing general understanding. In Milan, a greater proportion required assistance, though a few demonstrated more advanced conceptual knowledge.

### Social determinants recalled by participants

Students identified a broad range of determinants, grouped into macro-categories:

- Economic conditions (income inequality, employment, economic status)—22 mentions
- Physical environment (housing quality, air and water safety, exposure to hazards)—22 mentions
- Education—14 mentions
- Lifestyle and behaviors (physical activity, smoking, alcohol consumption)—13 mentions
- Biological/genetic factors—13 mentions
- Nutrition—9 mentions
- Social environment (community support, family role, discrimination)—8 mentions
- Access to healthcare—7 mentions

Some students also mentioned non-traditional factors, including age (6), sex (4), chronic health conditions (2), and life expectancy (2). Although not typically classified as SDH, these factors were perceived as indirectly influencing health through social, economic, and environmental interactions.


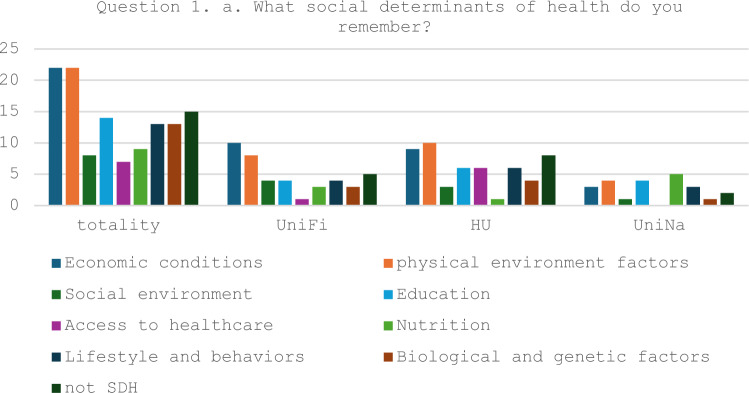


### Perceived impact of SDH on health

Students highlighted the influence of low socioeconomic and social status on health, associating it with poorer living conditions and higher exposure to disease risk. Common examples included respiratory infections related to pollution, unsafe housing, and smoking, as well as benefits of balanced diet and physical activity.

### Regional variations were observed

- University of Florence: emphasis on poverty and environmental health risks
- Humanitas University: focus on hygiene and smoking behaviors
- Naples: broader understanding of SDH as multifactorial contributors to health inequality

### Most important determinant

Overall, 27% of respondents identified socioeconomic status as the primary determinant, 24% cited education or diet, 13% housing, and 7% access to potable water or quality healthcare services.

Regional trends showed:

- University of Florence: 40% education, 40% socioeconomic status, 20% diet
- Humanitas University: 30% socioeconomic status, 30% housing, 20% access to healthcare, 20% education
- University of Naples Federico II: 50% diet, 20% access to potable water, 10% each for education, socioeconomic status, and housing

Students associating health with socioeconomic status emphasized unequal access to medical services and its influence on other determinants. Those prioritizing education noted its role in symptom recognition, preventive behavior, and informed decision-making. Diet was valued for chronic disease prevention and management, while housing and clean water were recognized for infection prevention.


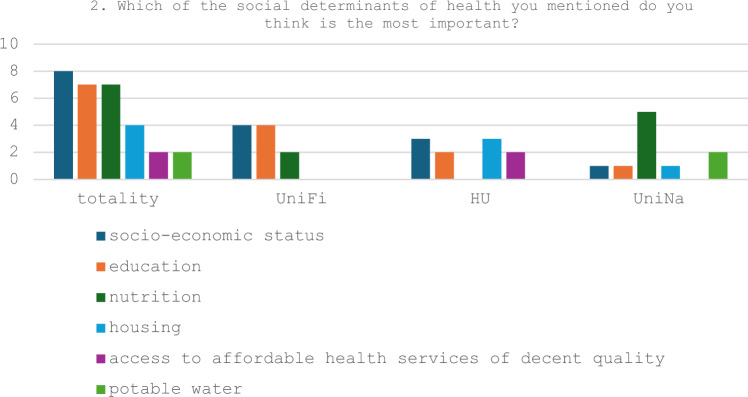


### Policy awareness and investment in SDH

Overall, students demonstrated limited awareness of national investments in SDH. Fifty-three percent believed investments were insufficient, 36.7% responded affirmatively but expressed skepticism about fund utilization, and 10% were unsure.

By region:

- University of Florence: 20% affirmative, 50% negative, 30% unsure
- Humanitas University: 20% affirmative (all skeptical of effectiveness), 80% negative
- University of Naples Federico II: 70% affirmative (43% skeptical), 30% negative

Half of the respondents were aware of at least one national campaign addressing SDH, commonly anti-smoking or cancer screening initiatives. A minority mentioned vaccination campaigns or activities linked to the National Recovery and Resilience Plan (PNRR).

Regarding healthcare funding, 43% believed hospitals received preferential investment, 37% disagreed, and 20% were uncertain. Milan students most frequently perceived hospital funding as dominant, while Florence students were evenly divided.


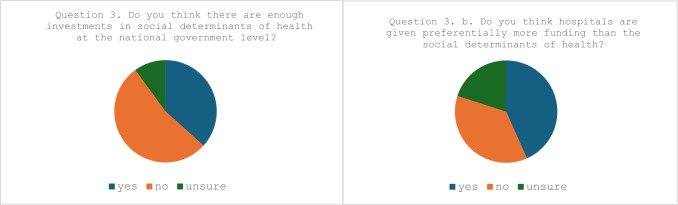


### Education and awareness of SDH in medical curricula

Sixty-seven percent reported insufficient SDH coverage in medical education. Affirmative responses (30%) were mainly from Florence, and 3% were unsure.

Eighty percent attended at least one lecture addressing SDH, often indirectly via public health or occupational medicine courses:

- University of Florence—90%
- Humanitas University—70%
- University of Naples Federico II—80%

Over 93% considered medical doctors responsible for advocating SDH, recognizing their authority and influence, though institutional support was deemed necessary for effective communication.


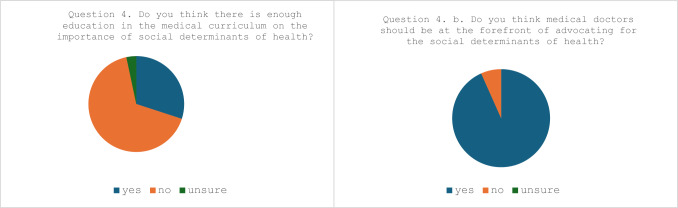


### Knowledge of international health frameworks

Awareness of the WHO Ottawa Charter (1986) was low: 70% had never heard of it, and only 12.5% of those familiar could recall its key elements. Florence students showed slightly higher awareness, while Milan and Naples had minimal exposure.

Similarly, 83% could not identify international goals, agreements, or treaties addressing SDH. Among the 17% who could, most cited environmental health campaigns, reflecting partial understanding of global health promotion efforts.

## Discussion

This study assessed the proficiency of final-year medical students from three Italian universities in understanding social determinants of health (SDH). By integrating qualitative thematic analysis with quantitative methods, the investigation explored students’ comprehension, perspectives, and awareness of the complex social, economic, and environmental factors influencing health outcomes.

The findings revealed a nuanced understanding of SDH among students, with notable variations across universities. While some demonstrated a solid foundational grasp, others struggled to articulate their knowledge, highlighting a potential gap between theoretical understanding and practical application. This applied understanding was assessed by examining students’ responses to open-ended questions in the questionnaire, specifically their ability to connect theoretical concepts of SDH to concrete examples affecting patient health and to propose strategies for addressing these determinants in clinical practice. For instance, students were prompted to describe how education, socioeconomic status, or nutrition might influence individual health outcomes and suggest interventions or preventive measures. This approach allowed the researchers to evaluate not only knowledge recall but also the reasoning process linking theory to practical scenarios**.** This gap may reflect current teaching methods, suggesting a need for innovative pedagogical approaches to enhance applied competency in SDH.

The study also examined students’ awareness of national government initiatives targeting SDH, revealing mixed perceptions regarding the adequacy of national investments. These discrepancies underscore the complexity of SDH policy implementation and the importance of further research to inform effective strategies.

Students demonstrated a growing interest in expanding their knowledge of SDH when prompted, recognizing the importance of mastering these concepts for clinical practice. The findings indicate that physicians, including specialists, should possess a strong understanding of SDH to address health outcomes effectively.

Additionally, patterns of shared perspectives observed among students within the same university suggest that their understanding of the social determinants of health (SDH) is influenced by common educational experiences within the institutional and educational context. Although this study did not include a formal curricular analysis, a descriptive consideration of publicly available curricula indicates that topics related to social determinants of health are generally incorporated within broader public health or hygiene courses, rather than delivered as dedicated modules explicitly structured around the SDH framework. This may lead to more fragmented or implicit exposure to these concepts, potentially affecting students’ ability to systematically integrate them into clinical reasoning. In light of these findings, the relevance of this study becomes particularly evident for the field of medical education. The students’ varied but limited ability to translate theoretical knowledge of SDH into practical clinical reasoning, underscores the need for curricula that explicitly train future physicians to incorporate social determinants into patient assessment.

Given that many students expressed a growing awareness of the importance of SDH and acknowledged that physicians should act as supporters for addressing the determinants within the wider society, the results highlight an opportunity for medical schools to strengthen this dimension of professional identity.

Therefore, this study emphasizes the importance of integrating structured SDH training into medical curricula, both to enhance clinical competence and to support the development of socially engaged physicians capable of advocating for health equity at individual, institutional, and societal levels.

## Conclusions

In conclusion, the study emphasizes the critical importance of integrating SDH education into medical curricula and promoting ongoing professional development. Enhancing students’ knowledge and awareness of SDH can support physicians in addressing the multifaceted determinants of health, advancing health equity, and fostering holistic approaches to patient care.

## Data Availability

Available from the corresponding author upon reasonable request.
